# A Combination of M50I and V151I Polymorphic Mutations in HIV-1 Subtype B Integrase Results in Defects in Autoprocessing

**DOI:** 10.3390/v13112331

**Published:** 2021-11-22

**Authors:** Jun Yang, Ming Hao, Muhammad A. Khan, Muhammad T. Rehman, Helene C. Highbarger, Qian Chen, Suranjana Goswami, Brad T. Sherman, Catherine A. Rehm, Robin L. Dewar, Weizhong Chang, Tomozumi Imamichi

**Affiliations:** 1 Laboratory of Human Retrovirology and Immunoinformatics, Frederick National Laboratory, Frederick, MD 21702, USA; jyang@mail.nih.gov (J.Y.); ming.hao@nih.gov (M.H.); chenq3@mail.nih.gov (Q.C.); suranjana.goswami@nih.gov (S.G.); bsherman@mail.nih.gov (B.T.S.); weizhong.chang@nih.gov (W.C.); 2 Virus Isolation and Serology Laboratory, Frederick National Laboratory, Frederick, MD 21702, USA; muhammad.khan@nih.gov (M.A.K.); trehman@mail.nih.gov (M.T.R.); hhighbarge@mail.nih.gov (H.C.H.); rdewar@mail.nih.gov (R.L.D.); 3 Laboratory of Immunoregulation, National Institute of Allergy and Infectious Diseases, Bethesda, MD 20892, USA; crehm@niaid.nih.gov

**Keywords:** polymorphisms, integrase, M50I, V151I, autoprocessing, maturation, viral replication

## Abstract

We have recently reported that a recombinant HIV-1NL4.3 containing Met-to-Ile change at codon 50 of integrase (IN) (IN:M50I) exhibits suppression of the virus release below 0.5% of WT HIV, and the released viral particles are replication-incompetent due to defects in Gag/GagPol processing by inhibition of the initiation of autoprocessing of GagPol polyproteins in the virions and leads to replication-incompetent viruses. The coexisting Ser-to-Asn change at codon 17 of IN or Asn-to-Ser mutation at codon 79 of RNaseH (RH) compensated the defective IN:M50I phenotype, suggesting that both IN and RH regulate an HIV infectability. In the current study, to elucidate a distribution of the three mutations during anti-retroviral therapy among patients, we performed a population analysis using 529 plasma virus RNA sequences obtained through the MiSeq. The result demonstrated that 14 plasma HIVs contained IN:M50I without the compensatory mutations. Comparing the sequences of the 14 viruses with that of the defective virus illustrated that only Val-to-Ile change at codon 151 of IN (IN:V151I) existed in the recombinant virus. This IN:V151I is known as a polymorphic mutation and was derived from HIVNL4.3 backbone. A back-mutation at 151 from Ile-to-Val in the defective virus recovered HIV replication capability, and Western Blotting assay displayed that the back-mutation restored Gag/GagPol processing in viral particles. These results demonstrate that a combination of IN:M50I and IN:V151I mutations, but not IN:M50I alone, produces a defective virus.

## 1. Introduction

Nascent human immunodeficiency virus type 1 (HIV-1) particles released from HIV-1-infected cells are immature and un-infectious viruses that contain immature Gag and GagPol polyproteins, accessory proteins including Nef precursor, and viral genomic RNAs [1]. The Gag and GagPol polyproteins are composed of non-functional precursors of viral structural proteins (matrix protein (MA), capsid protein (CA), nucleocapsid protein (NC) and the viral enzymes: protease (PR), reverse transcriptase (RT), RNase H (RH), and integrase (IN)). In the nascent particles, mature PR cleaves the Gag and GagPol polyproteins at 11 cleavage sites and one site on the Nef precursor and then cut off each functional protein in the particles [1,2,3]. Subsequently, the virus becomes an infectious virus. While the mechanism of these catalytic activities and regulation of the cleavages by the mature PR are well-described [4], the regulatory mechanism of initiation of the excision of an embedded immature PR in GagPol polyprotein is less investigated. It is reported that GagPol polyproteins initially dimerize and then, the embedded PR domain releases a mature dimerized PR [5,6,7]. This processing step is called autoprocessing and the initial autoprocessing is trigged at the cleavage site between P2 and NC in an intra-molecular (*cis*) manner [5], and the inhibition of the initial cleavage of GagPol polyproteins has been considered a unique therapeutic target [8,9].

Naturally occurring polymorphisms (NOP)s and genetic diversity in the HIV genome often regulate drug susceptibility, virus evolution, and viral infection/replication [10,11,12,13]. In a recent sub-study of the Strategic Timing of Antiretroviral Therapy (START) study [14], a Genome-wide association study (GWAS) was conducted using HIV RNA sequences of plasma virus from anti-retroviral therapy (ART) naïve patients. It was reported that 14 NOPs were identified as virus load (VL)-associated mutations [15]. In our previous study, the 14 variants encoding each mutation were constructed using HIVNL4.3 backbone, and the viral replication ability was compared among the mutations and wild-type virus in in vitro HIV replication assay [16]. Subsequently, we found that a mutant containing Met-to-Ile change at codon 50 in IN (IN:M50I) was a defect in infection/replication caused by interruption of the initiation of autoprocess step; however, due to the coexisting other NOPs, Asn-to-Ser change at codon 79 in RH (RH:N79S), or Ser to Asn mutation at codon 17 in IN (IN:S17N), the impaired replication was restored. Therefore, although the molecular mechanism of suppressing the initiation step is still unclear, we concluded that IN:M50I mutation is a lethal mutation, and RH:N79S and IN:S17N mutations are compensatory mutations for IN:M50I mutation. Although these mutations: IN:M50I, RH:N79S and IN:S17N, were reported NOPs mutations [11,12,15], we presumed that a particular ART might facilitate the emergence of the mutations and affect susceptibility to integrase inhibitor, since IN:M50I is reported as a mutation associated with resistance to dolutegravir (DTG), which enhances resistance of a mutant carrying R263K mutation to DTG, but not resistance to the drug by itself [17]. If a particular treatment which increases frequency of the emergence of M50I were identified, it would have a clinical advantage in designing therapies targeting the integrase. In this study, we initially aimed to clarify the presence of the three NOP mutations in the clinical isolates from HIV-infected patients in NIAID clinical trials and elucidate whether there was an emergence of the mutations during ART and the correlation between the defect and the compensatory mutations. We endeavored to define a population of each mutation in 529 clinical isolates in the trials. We expected that variants containing the IN:M50I mutation should possess at least one of the compensatory mutations; however, 14 samples in the 529 isolates encoded only IN:M50I change. This observation prompted us to identify the other compensatory mutation in the 14 viruses. We report here that Val-to-Ile mutation at codon 151 of IN, IN:V151I, plays a key role in the defect in the initiation of autoprocess step.

## 2. Materials and Methods

### 2.1. Ethics Statement

Approval for these studies, including all sample materials and protocols, was granted by the National Institute of Allergy and Infectious Diseases (NIAID) Institutional Review Board, and participants have informed a written consent prior to blood being drawn. All experimental procedures in these studies were approved by the National Cancer Institute at Frederick and Frederick National Laboratory for Cancer Research (the protocol code number: 16–19, approval data: 6 January 2017).

### 2.2. HIV RNA Sequencing

All HIV RNA sequences were derived plasma samples from 209 HIV-infected patients in NIAID clinical trials. The plasma samples were obtained from pre, on, or post anti-viral therapy (ART) with or without integrase inhibitors. Total analyzed time points per patient were donor dependents (the blood drawing points were from 1 to 14 times) (Table S1). HIV viral RNA from patient plasma was extracted from the plasma using the EZ1 Advanced XL instrument and EZ1 Virus Mini kit v2.0 (cat# 955134, Qiagen, Germantown, MD, USA). The extracted RNA was used for one-step reverse transcription-PCR amplification (RT-PCR) using PrimeScript™ One Step RT-PCR Kit V.2 (cat# RR055, Takara, Rockville, MD, USA) using HIV-specific PCR primers (E+ primer and A4.5 kb primer sets) (Table S2) to amplify the whole HIV *gag* or *gag-pol* regions. The reaction was performed in three steps: 1st step: 60 °C for 1 hr, 94 °C for 2 min; 2nd step: 60 cycles at 94 °C for 1 min, 61 °C for 30 s and 72 °C for 4 min 45 s; and 3rd step: 1 cycle of 72 °C for 10 min and then hold at 4 °C. The E+ primer set resulted in a 4482 bp product (HXB2: 737-5219 nt). The A4.5 kb primer set resulted in a ~4537 bp product (HXB2: 682–5219 nt). Quality of the PCR products was analyzed by agarose gel and the appropriate size of products was purified, and then PCR products were quantified using Qubit ds DNA specific assay kit (Thermo Fisher, Waltham, MA, USA). For the next generation sequence (NGS), Illumina MiSeq instrument (Illumina, San Diego, CA, USA) along with Nextera XT DNA Library Preparation Kit (cat# FC-131-1024) (Illumina), MiSeq Reagent Nano Kit v.2, 500 Cycles (cat# MS-103-1003) (Illumina), and Nextera XT Index Kit (cat# FC-131-1001) (Illumina) were used. Briefly, 1 ng of the purified amplicons were used for the Illumina MiSeq enzymatic based Tagmentation reaction that fragments and adds (tags) partial adapter sequences at the ends of amplicons. Nextra XT PCR was performed to add the index and adapter sequences required for cluster formation on the flow cells during the sequencing run. The NGS library products were cleaned up, normalized, and then treated with NaOH followed by the equimolar sample pooling in a single tube. The final products were loaded to the MiSeq cartridge and MiSeq sequencing run was performed following a standard protocol from the vendor.

### 2.3. Sequence Assembly 

Sequence results from the MiSeq were assembled as previously described [18]. In brief, Lastal (last-490, Built on 7 October 2014) [19] was used to filter paired reads for HIV-specific sequences from the sequencing data using HXB2 sequence as a reference. Filtered reads were then adapted and quality trimmed with Trimmomatic (v0.33) [20] and the remaining reads were de novo assembled using Trinity (r2011-11-26) [21]. Resulting contigs from this assembly were ordered and merged using V-FAT (v1.1, https://www.broadinstitute.org/viral-genomics/v-fat, accessed on 28 September 2021) with HXB2 reference backbone to produce an initial sample-based consensus sequence. NovoAlign (v.3.02.12, http://novocraft.com, accessed on 28 September 2021) was used to align the paired reads to this initial consensus sequence. More than 200× depth coverages for all regions of interest were required for further analysis. The criteria removed any false codons in the sequences. The above steps were executed through wrappers in viral-ngs (v1.0.0-4, https://github.com/broadinstitute/viral-ngs, accessed on 28 September 2021). A pileup file was then produced from the initial consensus alignment using SAMtools mpileup (v0.1.19) [22] and a variant read count file was created using VarScan (v2.4.2) [23] from the pileup. Based on the VarScan output, a final consensus sequence was created as follows. For calling the consensus sequence, at each position, we called the base with the highest frequency of reads supporting the call at that position. If more than one variant has the same frequency at a given position, we call an ambiguous base following the IUPAC code (https://www.bioinformatics.org/sms/iupac.html, accessed on 28 September 2021). We did not focus on minor variants for this study as our goal was to define the combination IN:M50I with a compensatory mutation.

On each sequencing run, we included one negative and one wild-type positive control. Both controls followed the same bioinformatic process for QC, assembly and final consensus calling. Either a negative control leading to an HIV consensus sequence or a positive control producing an unexpected consensus sequence led to a failure of the sequencing run.

### 2.4. Cells

Peripheral blood mononuclear cells (PBMCs) were isolated from healthy donors’ apheresis packs using a lymphocyte separation medium (ICN Biomedical, Aurora, OH, USA) [24], CD4(+) T cells were purified from PBMCs using CD4 MicroBeads (Miltenyi Biotec, Auburn, CA, USA) according to the manufacturer’s instructions. The purity of the cell types was at least 90%, based on flow cytometric analysis. Cell viability was determined using the trypan blue (Thermo Fisher) exclusion method. HEK293T cells were obtained from ATCC (ATCC, Manassas, VA, USA) and maintained in complete D-MEM (Thermo Fisher) supplemented with 10 mM 4-(2-hydroxyethyl)-1-piperazineethanesulfonic acid (HEPES) pH 7.4 (Quality Biological, Gaithersburg, MD, USA), 10% (*v*/*v*) fetal bovine serum (FBS; Thermo Fisher), and 50 μg/mL gentamicin (Thermo Fisher) as previously described [24,25].

### 2.5. Construction of Plasmids Encoding HIV Variants 

Plasmids encoding HIV variants were constructed by inducing the mutations on pNL4.3 [26] (the plasmid was obtained from M. Martin through the AIDS Research and Reference Reagent Program, National Institute of Allergy and Infectious Diseases, National Institutes of Health) using the QuickChange Lightning kit (Agilent Technologies, Santa Clara, CA, USA) with mutagenesis primers (Table S2) as previously described [16]. Mutagenesis was confirmed by Sanger DNA sequencing using the BigDye terminator v.3 (Thermo Fisher) with SeqStudio Genetic Analyzer (Thermo Fisher), and plasmids were maintained in Stbl3 *E. coli* (Thermo Fisher). The plasmid purification was carried out using the EndoFree Plasmid Maxi Kit (Qiagen).

### 2.6. Recombinant HIV-1 Viruses

Recombinant HIV-1 variants were prepared by transfection of the pNL4.3 mutants into HEK293T cells using TransIT-293 (Mirus, Houston, TX, USA) and Opti-MEM I medium (Thermo Fisher) following a method previously reported [16]. Culture supernatants were collected at 48 h after transfection and then filtrated through 0.45 μm pore size filter membranes (MiliporeSigma, Burlington, MA, USA). Virus particles in the filtrate were pelleted by an ultra-centrifugation on 20% (*w/v*) sucrose (MiliporeSigma) in 10 mM HEPES-150 mM NaCl buffer (pH 7.4) as previously described [16,27]. Viral pellets were resuspended in PBS and stored at −80 °C until use. Concentration of HIVp24 antigen in each stock was determined by a p24 antigen capture kit (PerkinElmer, Waltham, MA, USA) and the concentration of total virus proteins were determined by a BCA protein assay kit (Thermo Fisher) [16,27].

### 2.7. HIV Replication Assay

Replication capability of each variant were determined using primary CD4(+) T cells as previously described [16]. CD4(+) T cells were stimulated with 5 μg/mL phytohemagglutinin (PHA; MiliporeSigma) in complete RPMI-1640 (Thermo Fisher) supplemented with 10 mM HEPES, 10% (*v*/*v*) FBS, and 50 μg/mL gentamicin (RP10). The PHA-stimulated CD4(+) T cells (10 × 10^6^ cells) were infected with 10 ng of p24 of each HIV variant at 10 × 10^6^ cells/mL in RP10 for two hours at 37 °C. The infected cells were washed with RP10, and then cultured at 1 × 10^6^ cells/mL in the medium in the presence of 20 units/mL of recombinant IL-2 (MiliporeSigma) for 14 days at 37 °C in T25 flasks [16,28]. Half of the cell-free culture supernatants were exchanged with fresh RP10 with IL-2 every 3 or 4 days of incubation. HIV-1 replication activity was determined by measuring p24 antigen levels in the culture supernatants using the p24 antigen capture assay [16].

### 2.8. Western Blotting

To confirm autoprocessing of Gag and GagPol polyproteins in HIV virions, Western Blotting (WB) was performed as previously described [16]. Due to abnormal sizes of M50I mutant (the diameters of the mutant particles were 190~300 nm, while those of Wt were 110~130 nm, and in this current study, the mutant previously used is renamed mNL(IN:M50I) as described in Section 3.1 below) with a defective autoprocessing of Gag and GagPol polyproteins (lacking of the cleaved mature forms of MA (p17), CA (p24), PR, RT, and IN in the virus particles) [16], we could not find an appropriate internal control for WB. Thus, we used the same amount of viral protein to demonstrate an impaired autoprocessing by WB. Briefly, total of 1 µg of viral proteins from HIV(WT) and HIV variants were separated in NuPAGE 4–12% Bis-Tris gels (Thermo Fisher) in MOPS running buffer (Thermo Fisher), and WB was performed using Rabbit polyclonal anti-Anti-HIV1 p55+p24+p17 antibody (Cat# ab63917, Abcam, Waltham, MA, USA). Protein bands were detected by using the ECL assay system [16]. 

### 2.9. Homology Modeling of HIV-1 Integrase

A full-length structure of IN was predicted using the currently available crystal structures of IN data from the protein database, as previously described [16]. To predict IN:M50I and IN:V151I structure in IN and RH fusion protein, an assembled structure was also constructed using AIDA program [29]. A detailed method is described in the Supplementary Methods.

### 2.10. Statistical Analysis

Intergroup comparisons were performed using two-tailed unpaired *t*-tests using Prism 8 software (GraphPad, San Diego, CA, USA). *p* values < 0.05 were considered statistically significant. To determine significant differences among a correlation between viral load and mutations, one way ANOVA test was conducted using Partek software (Partek St. Louis, MI, USA).

## 3. Results

### 3.1. Population Analysis of M50I in NIAID Clinical Samples

**Figure 1:**
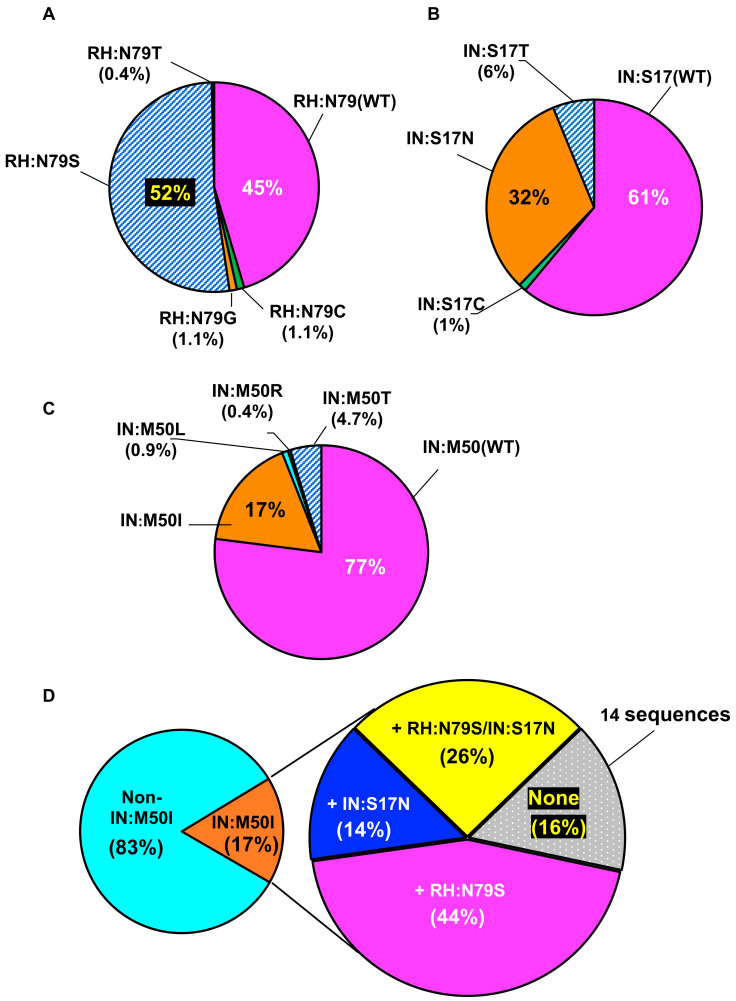
Population analysis in NIAID clinical isolates. (**A**–**D**) The emergence of the RH:N79S, IN:S17N, and IN:M50I polymorphism in NIAID clinical trials. HIV RNA sequences of 529 clinical isolates from 209 patients were analyzed, focusing on RH and IN genes. (**A**–**C**) The population analysis were performed at RH:N79 (**A**), IN:S17 (**B**), and IN:M50 (**C**). Data indicate % of the population for each mutation. (**D**) A dissertational analysis of sequence containing IN:M50I. The sequences containing IN:M50I mutation were further analyzed to determine the population in the combination of mutations. None indicates no combination with either RH:N79S or IN:S17N.

Our previous study using a series of recombinant HIV-1NL4.3 mutants demonstrated that virus containing IN:M50I mutation, mNL(IN:M50I), was a defective virus that suppressed the initial cleavage of GagPol polyproteins and viral release (note: the defective recombinant virus was depicted HIV(IN:M50I) in the previous work [16]; however, to avoid confusion, the mutant is redesignated as mNL(IN:M50I) in the current study); RH:N79S and IN:S17N mutations rescued the defect [16]. In order to define the emergence of those mutations during ART in NIAID clinical trials and the correlation between the defect and the compensatory mutations, a viral population analysis was conducted focusing on three mutations. We initially obtained a total of 556 HIV RNA sequence data from an NIAID HIV sequence data storage. Those sequences were derived from 213 patients enrolled in different clinical trials protocols with or without integrase inhibitors. HIV sequence reactions were conducted using plasma containing >1000 copies/mL of HIV RNA. After initial quality analysis, we excluded 27 sequences from four patients that contained integrase inhibitor (INI)-resistant mutations (Y143C/H/R; Q148H/K/R or N155H/S) [11,12]. A remaining total of 529 sequences from 209 patients (Table S1) were subjected to the population analysis focusing on RH:N79S, IN:S17N and IN:M50I that exist in majority of these sequences. First, we investigated the emergence of every single mutation in the amplicons. As shown in Figure 1A–C, RH:N79S, IN:S17N, and IN:M50I mutations were detected 52% (275 amplicons), 32% (169 amplicons), and 17% (90 amplicons), respectively.

We next dissected the 90 amplicons containing IN:M50I, to define the population of combination; the combinations of RH:N79S/IN:M50I and IN:S17N/IN:M50I were detected in 40 (44%) and 13 (14%) amplicons, respectively, and RH:N79S/IN:S17N/IN:M50I was detected in 26% (23) amplicons (Figure 1D). Surprisingly, 16% (14 amplicons) possessed IN:M50I alone without RH:N79S or IN:S17N compensatory mutation (Figure 1D). Those 14 sequences were derived from eight patients, and the range of VL of the patients was 1207–201,353 copies/mL (Table 1). Since IN:M50I alone conferred a defective virus in GagPol processing with the suppression of virus release in our previous work [16], we presumed that the virus encoding only IN:M50I should contain uncharacterized compensatory mutation(s) other than the compensatory mutation (RH:N79S or IN:S17N), thus they may be released from cells with similar levels to other mutations (Table 1). To define whether a correlation between the VL range and M50I mutations is significant or not, we performed a statistical analysis using one way ANOVA unpaired method. It is reported that the three mutations are NOPs [11,12,15]; thus, we disregarded the treatment regimens in the initial analysis. The results demonstrated that among M50I alone vs. M50I/N79S, M50I vs. M50I/S17N, M50I vs. N79S/S17N, M50I vs. non-M50I, M50I vs. non-M50I/N79S, non-M50I/S17N, non-M50I vs. N79S/S17N, there are no significant differences in VL (Figure S1), indicating that VL is not correlated with M50I mutations. 

### 3.2. Analysis of Sequence Alignments 

**Figure 2:**
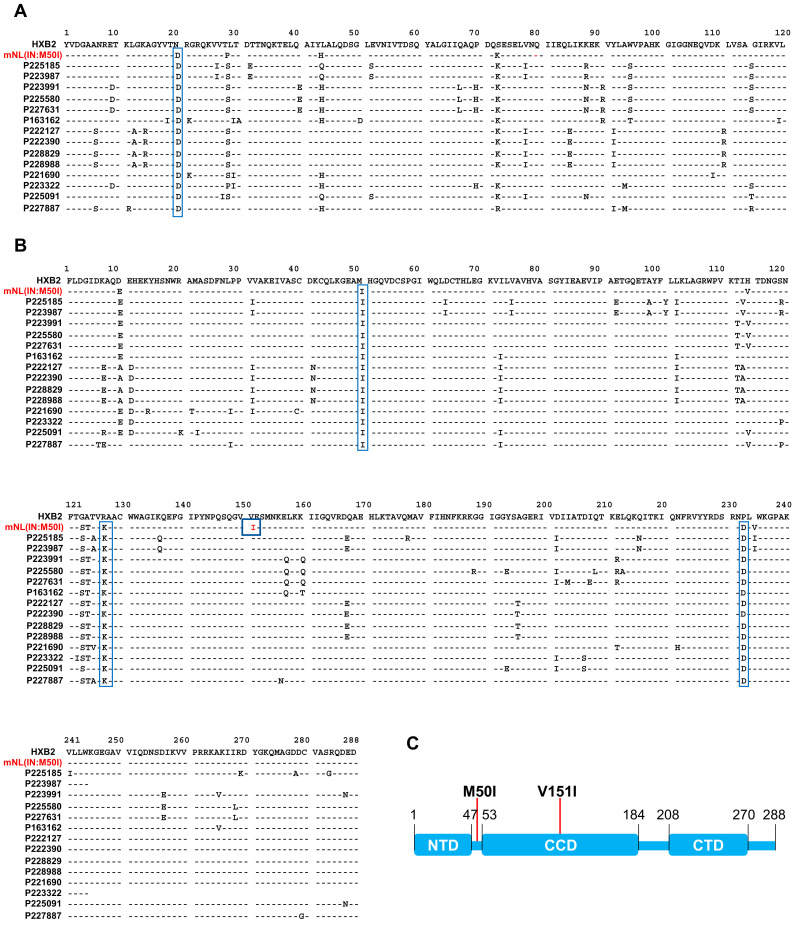
Amino acid Sequence Alignment of RH and IN. Amino acid sequences of RH (**A**) and IN (**B**) of 14 clones containing IN:M50I were aligned using the HIValign tool in LANL program (https://www.hiv.lanl.gov/content/sequence/VIRALIGN/viralign.html, accessed on 28 September 2021). HXB2 sequence was used as a reference sequence in the alignment. The mNL4.3 sequence was also included in the alignment as a control. (**C**) A schematic diagram presents mature HIV-1 IN. IN is composed of the N-terminal domain (NTD) (codons 1–49), the catalytic core domain (CCD) (codons 50–212) and the C-terminal domain (CTD) (codons 213–288) [30,31,32,33].

We next endeavored to identify the unknown compensatory mutation in the 14 viruses containing M50I mutation alone. To identify the compensatory mutation(s) using the HIVHXB2 sequence as a reference sequence, we compared the 14 sequences from alignments (Figure 2A,B). The clinical profiles of samples were also compared among the 14 samples from 8 donors. Two donors were ART naïve, and one donor was initially under the Long Term Non-Progressor protocol but was later recognized as a viremic patient, then moved to under ART from 2019 (Table S3). Based on the treatment histories, it appears that IN:M50I was detected in a treatment independent manner. The defective virus sequence in our previous study, mNL(IN:M50I) [16], was also included in the alignment (note: as described above, HIV(IN:M50I) in the previous work is renamed mNL(IN:M50I) in the current study). The mNL(IN:M50I) virus was constructed using a modified HIVNL4.3 strain (mNL). We assessed for the mutation(s) that are commonly expressed in the 14 sequences but not in mNL(IN:M50I). In the clinical samples, RH:N20D, IN:M50I, IN:R127K, and IN:N232D were common mutations (Figure 2A,B). However, those mutations were also present in mNL(IN:M50I). Therefore, there was no unique common mutation among the 14 clinical isolates. Intriguingly, IN:V151I mutation appeared in the defect recombinant virus but not in clinical samples (Figure 2B). IN is composed of three domains: the N-terminal domain (NTD), the catalytic core domain (CCD), and the C-terminal domain (CTD) (Figure 2C). The codons 50 and 151 are located on the hinge region between NTD and CCD, and in CCD, respectively (Figure 2C and Figure S2) [30,31,32]. Therefore, it was speculated that in the immature IN in GagPol polyprotein, both residues might interact and induce a defective virus.

### 3.3. Evaluation of the Impact of IN:V151I Mutation on HIV Replication and Autoprocessing

To delineate whether our hypothesis, that a combination of IN:M50I and IN:V151I causes a defective virus, was correct or not, we compared virus replication capability using a series of mutants. In the current study, we constructed the mutants utilizing the clones that we have previously used [16]. As mentioned above, the constructs derived from mNL4.3 backbone contain the endogenous IN:V151I mutation. Therefore, the mutation was back-mutated from Ile-to-Val by site-directed mutagenesis on the previously used constructs. The resulted constructs, p(WT), p(IN:M50I alone), p(IN:V151I alone), and p(IN:M50I/V151I) were transfected into HEK293T cells, and then viral stocks were prepared as described in the Materials and Methods [16]. Pelleted viral particles were resuspended in PBS (1/100 volume of the starting materials) and used as viral stocks. To determine the amounts of virus in each stock, we measured HIV p24 antigen concentrations in each stock. HIV containing IN:M50I alone or IN:V151I alone demonstrated comparable amounts of p24 concentrations with that of HIV(WT) (110.0 ± 22.7 μg/mL, *n* = 4) (Table S4). In striking contrast, the p24 concentrations of HIV(IN:M50I/V151I) stocks were 0.30 ± 0.1 μg/mL (*n* = 4) and thus around 0.3% of that in HIV(WT) (*p* < 0.01), indicating that the combination of mutation suppressed virus release. Since we were not able to produce a high TCID50 titer of virus stock of HIV(IN:M50I/V151I), we infected 10 × 10^6^ cells of primary CD4(+) T cells with 10 ng of p24 amounts of virus for viral replication assay as described in the Materials and Methods. 

**Figure 3:**
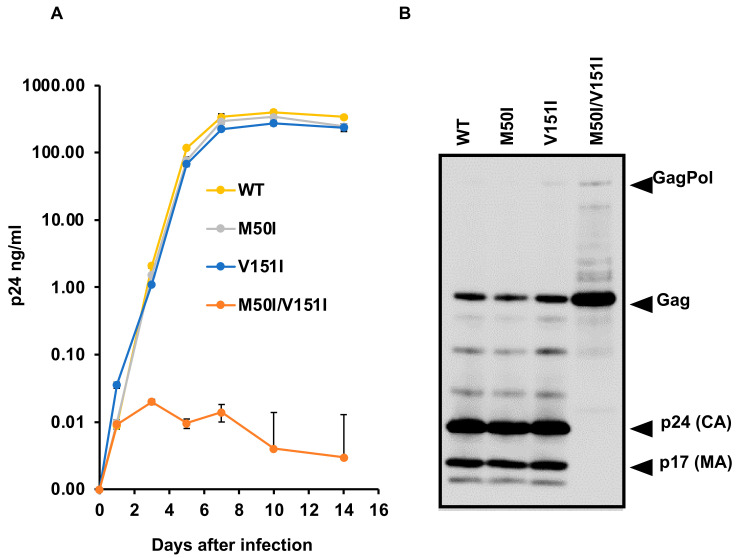
Characterization of IN:V151I Effect on HIV Replication: (**A**) Comparison of HIV replication capability. PHA-stimulated primary CD4(+) T cells from healthy donors were infected with 10 ng p24 amounts of HIV(WT) or variants containing mutation V151I, M50I, and M50I/V151I. The infected cells were cultured for 14 days with media changed every 3–4 days. HIV replication was monitored using a p24 antigen capture kit (PerkinElmer). Representative data from three independent assays are presented as mean ± SD (*n* = 3). (**B**) A total of 1 μg of total viral particle proteins from HIV(WT) and HIV variants were subjected for WB using polyclonal anti-Anti-HIV1 p55 (Gag)+p24(CA)+p17(MA) antibody (Cat# ab63917, Abcam) as described in the Materials and Methods.

As shown in Figure 3A, HIV(IN:M50I) and HIV(IN:V151I) single mutation replicated at rates comparable with HIV(WT) in primary CD4(+) T cells from day one after infection; however, a variant containing both mutations exhibited impaired replication. The combination led to a replication-incompetent virus (0.01% of replication capability compared to HIV(WT), *p* < 0.001, *n* = 5), while HIV(IN:M50I) and HIV(IN:V151I) replicated 89 ± 6.5% (*n* = 5, *p* = 0.795) and 90 ± 3.8% (*n* = 5, *p* = 0.751) of HIV(WT) at day 7, respectively. These data indicated that the defective recombinant virus in the previous report of HIVmNL(IN:M50I) was caused by the combination of IN:M50I and IN:V151I mutations.

To confirm the combination effect of the mutations, we carried out WB analysis using 1 μg of total viral proteins of each virus with anti-p55(Gag)+p24(CA)+p17(MA) antibody to confirm that the single mutation had no impact on the GagPol processing. Mature cleaved p17 and p24 fragments were detected in HIV(WT), HIV(M50I), and HIV(IN:V151), but not in HIV(IN:M50I/V151I), and accumulation of uncleaved GagPol polyprotein was detected in the mutant (Figure 3B) as we showed in previous work [16]. Thus, the combination of mutations resulted in a defective HIV, and since all 14 clinical isolates lacked IN:V151I mutation and were identified in plasma containing 1207-201,353 copies/mL (Table 1), this indicated that the virus is released from cells. However, they might be defective in reinfection by uncharacterized mechanism(s), such as immature or lack of other viral proteins. We need to study further to conclude the M50I effect on replication capability in clinical isolates. 

### 3.4. Population Analysis of the Combination of IN:M50I/IN:V151I in HIV Sequence Database

To elucidate the population diversity of HIV carrying the IN:M50I/IN:V151I mutant in the plasma virus, we compared HIV sequences focusing on the mutations using NIAID and the Los Alamos National Laboratory (LANL) HIV database. The results demonstrated that the combination was not detected in the total 529 sequences samples from 209 patients in NIAID samples (Table 2); however, in the LANL HIV database, 0.43% (27 sequences) of subtype B and 0.1% (3 sequences) of subtype C contained the combination, and other subtypes did not have HIV sequences with the combination (Table 3). 

**Figure 4:**
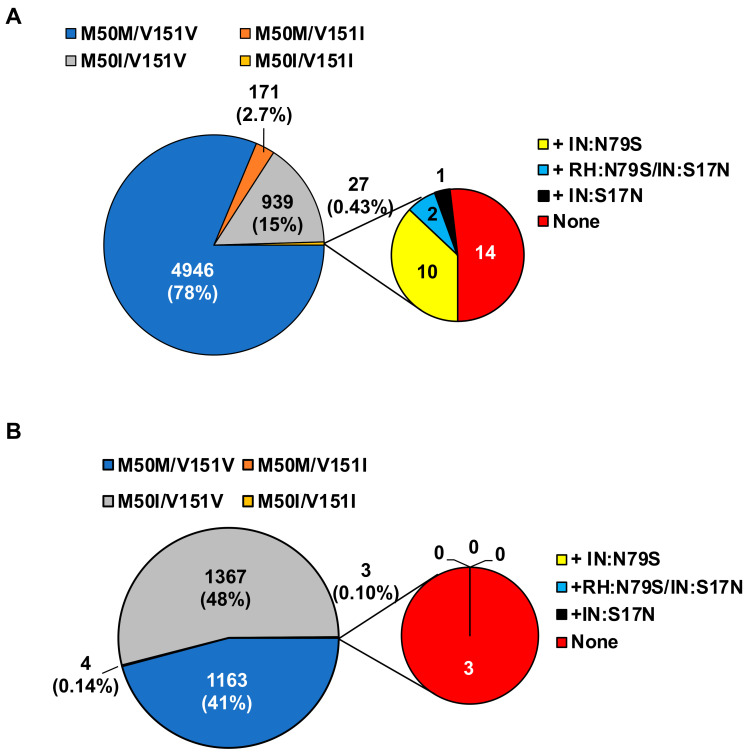
Population diversity of a combination of IN:M50I/IN:V151I mutations in subtype B and C HIV in the LANL HIV database. Population analysis was conducted to determine the diversity of HIV sequences containing IN:M50I and IN:V151 in (**A**) subtype B and (**B**) subtype C in the LANL HIV database. All HIV-1 subtypes of sequences were downloaded from the LANL.GOV website. The sequences containing IN (P31) regions were selected and only one sequence per patient was used in the population analysis. As shown in Table 3, total 27 and 3 of HIV sequences contained the lethal combination in subtype B and C, respectively. The sequences containing the defective mutations were further analyzed to define a population of RH:N79S and IN:S17N.

As described above, the virus replication assay indicated that an HIV containing the combination of M50I/V151I without RH:N79S or IN:S17N was a replication incompetent virus. Thus, we further analyzed the viruses containing the combination focusing on the presence of the compensatory mutation. In subtype B, nearly half of the sequences contained either RH:N79S or IN:S17N mutation, and surprisingly, 14 out of the 27 sequences did not contain the compensatory mutations (Figure 4A), and in subtype C, none of the three sequences possessed RH:N79S or IN:S17N mutations (Figure 4B). 

## 4. Discussion

We recently reported that the presence of IN:M50I in HIV-1 suppresses virus release and inhibits HIV maturation by interrupting the initiation of autoprocessing; subsequently, the virus is impaired in replication, and other mutations, RH:N79S or IN:S17N, restored the viral replication [16]. In the current work, we found that the defect in GagPol autoprocessing resulted from the presence of a combination of IN:M50I and IN:V151I, not by IN:M50I alone.

IN:M50I and IN:V151I mutations are naturally occurring SNPs and are reported as mutations associated with resistance to integrase inhibitors [17,34,35], IN:M50I is selected during the integrase strand transfer inhibitor (INSTI), DTG [34,36], and raltegravir [35] therapy as a resistance-associated mutation; however, the mutation perse has no impact on resistance to IN inhibitors [17], only a combination of the mutation with drug resistance mutation increases resistance [11,12,17,34,35,37,38,39,40,41,42]. 

IN:V151I was an endogenous mutation in the HIVNL4.3 strain. Therefore, before the drug treatment, if HIV RNA sequence data indicated that IN:V151I is present in the HIV without IN:S17N or RH:S79S, IN:M50I selection during the treatment may be delayed because the combination is a suicide mutation for HIV, and the treatment effect may last longer. However, further study needs to define the molecular mechanism of the defect, since population analysis of the LANL HIV database indicated that 14 of 27 sequences in subtype B and 3 sequences of subtype C contained IN:M50I/V151I mutation without encoding RH:N79S or IN:S17N. In our previous work, HIV containing IN:M50/V151I mutants without RH:N79S or IN:S17N was defective, and the virus release and replication were significantly suppressed [16]; it is reasonable to assume that the uncharacterized compensatory mutations other than RH:N79S and IN:S17N in IN:M50I/IN:V151I mutants may exist (Figure 4B,C), but this needs further study. Identifying the compensatory mutation(s) may reveal a new drug target to develop INSTI-resistant viruses. 

**Figure 5:**
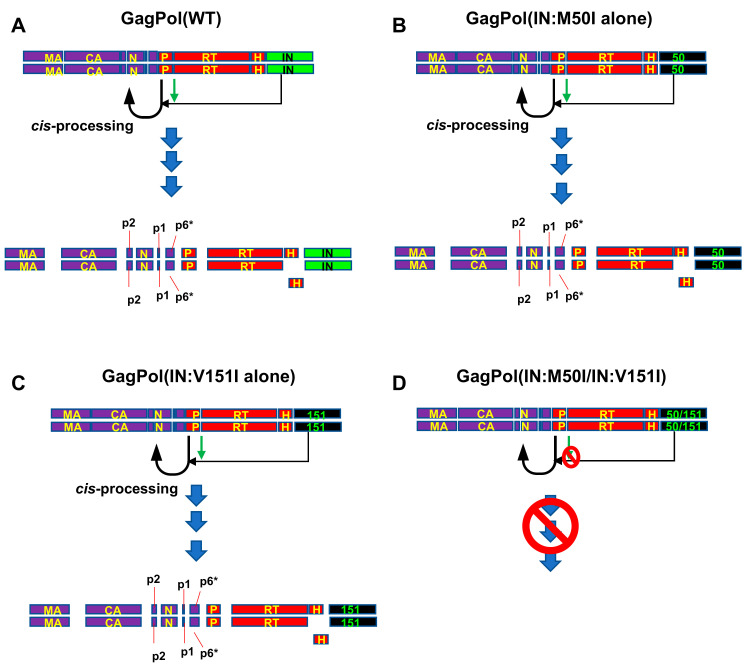
A schematic diagram illustrating IN:M50I/V151I effect. (**A**–**C**) GagPol polyproteins homodimerized, and the embedded PR in GagPol(WT) (**A**), GagPol(IN:M50I) (**B**), or GagPol(IN:V151I) (**C**) cleaves the polyproteins at the cleavage site between P2 and NC as the initial processing [5] and then sequential processing occurs. It is reported that IN positively regulates the initiation of autoprocessing [44,45,46] and the cleavage site between PR and RT is involved in the regulation (as shown in green arrows) [16]. (**D**) GagPol(IN:M50/IN:V151I) mutations also form homodimers [16]; however, the autoprocessing step is inhibited by an uncharacterized mechanism(s). Green arrows: the cleavage site between PR and RT regulates autoprocessing [16]. The region negatively regulates the autoprocessing in the presence of IN:M50I/V151I mutation via an uncharacterized mechanism. MA: matrix proteins (p17), CA: capsid protein (p24), N: nucleocapsid protein, P: protease, RT: reverse transcriptase, H: RNase H, IN: integrase, 50: M50I change in IN, 151: V151I change in IN.

GagPol polyproteins are cleaved by mature PR in viral particles [5,6,7]. The embedded PR in GagPol is an immature protein. However, the embedded PR triggers the initial process of cleavage through *cis*-interaction [5] (Figure 5A). The initial cleavage site locates between P1 and nucleocapsid protein (N) (Figure 5A). This processing step, so-called autoprocessing, is suppressed by truncated IN [43,44], an IN inhibitor [45], or the emergence of mutations (T124N/T174I) associated with resistance to an allosteric IN inhibitor (ALLINI) in IN [46], indicating that IN positively regulates the processing (Figure 5A). Taken together with our previous report (IN:M50I mutation inhibits the initiation of autoprocessing: the virus particles lack cleaved Gag and GagPol proteins [16]), the suppression was caused by the combination of IN:M50I and IN:V151I (Figure 5D) but not by a single mutation of IN:M50I or IN:V151I (Figure 5B,C). 

A complete IN structure model is not available; thus, we created a predicted model by in silico modeling system. The codon 151 does not locate on IN surface (Figure S2A), and a predicted distance between codon 50 and 151 was 19.2 Å (Figure S2B). In our previous work, we reported that the autoprocessing is regulated by not only IN but also the cleavage site sequence between PR and the reverse transcriptase (RT) (Figure 5D) [16]. Therefore, the regulation of the autoprocessing may be complex in protein interaction among the cleavage site sequence, IN:M50 and IN:V151, and the compensatory mutations of RH:N79S and IN:S17N. It is reasonable to presume that the structure of immature IN in GagPol may differ from the investigated mature forms of IN. The structure analysis of IN in GagPol may shed new light on the study for regulation of autoprocessing. In conclusion, this study shows that the M50I defective virus which suppressed the initiation of autoprocessing of GagPol polyprotein in our previous work [16] is a result of the presence of a combination of M50I and V151I mutations in IN. Therefore, endogenous mutations in IN or the HIV genome may influence the function of IN in regulation of the autoprocessing [16,45,46]. It is reported that the enzymatic activity of IN with M50I mutation is comparable to that of IN(WT) [17]; however, to precisely compare the function of IN with M50I alone, V151I alone, and the combination of M50I and V151I mutations, we need the enzymatic study using recombinant IN. Further studies may provide new insights into the development of novel therapies targeting autoprocessing/maturation steps [4,8,9].

## Figures and Tables

**Table 1:** A profile of CD4(+) T cell counts and VL among patients infected with HIV containing IN:M50I mutation.

IN:50 *	RH:79 *	IN:17 *	Sequence #†	Patient #‡	T.CD4 (Cells/μL) **	VL (Copies/mL)
M50M ***	N79N	S17S	139	55	3–227	874–2,121,990
M50I	N79N	S17S	14	8	222–705	1207–201,353
M50I	N79N	S17N	13	3	174–940	2024–741,044
M50I	N79S	S79S	36	15	110–734	1529–1,009,970
M50I	N79S	S17N	23	7	18–589	4677–977,945
M50I	N79S	S17C	2	1	46–52	786,337–834,397
M50I	N79S	S17T	2	1	452–476	3337–4893

*: Amino acid residues at each codon: codon 50 in IN (IN:50), codon 79 in RH (RH:79), and codon 17 in IN (IN:17), were compared with that in consensus B sequence. In the consensus B sequence, IN:50, RH79, and IN:17 are methionine (IN:M50), asparagine (RH:N79), and serin (IN:S17), respectively; **: total CD4(+) cells count per microliter. ***: aa changes compare to the consensus sequence. Single letter code of amino acid; M: methionine, I: isoleucine, N: asparagine, C: cysteine, T: threonine. †: numbers of sequenced samples, ‡: patient numbers.

**Table 2:** Population analysis of IN:V151 variation in NIAID samples.

	IN:50 *						
		M50I **	M50L	M50M	M50R	M50T	Samples/Patients ^#^
IN:151 *	V151I	0 ^##^	0	3/2	0	11/2	14/4 (2%) ☨
	V151V	90/35	5/2	404/161	2/2	14/5	515/205 (98%)
	Sample ^#^	90/35	5/2	407/163	2/2	25/7	529/209 (100%)

*: Amino acid residues at each codon: codon 50 (IN:50) or codon 151 in IN were compared with that in consensus B sequence. In the consensus B sequence, AA at IN:50 and IN:151 are methionine (M) and valin (V), respectively. **: Single letter code of amino acid; M: methionine, I: isoleucine, L: leucine, R: arginine, T: threonine, #: analyzed sample numbers of sequence/patients, ##: sequence numbers containing the mutation, ☨: numbers in parentheses indicate population percentages of the mutants.

**Table 3:** Population diversity of M50I or V151I in the LANL HIV database *.

Subtype	M50I	V151I	M50I/V151I	Total **
A	10.89%	0.00%	0.00%	1194
B	15.27%	3.22%	0.43%	6335
C	47.69%	0.31%	0.10%	2871
D	1.54%	0.77%	0.00%	260
F	10.42%	1.39%	0.00%	144
G	15.97%	0.69%	0.00%	144
H	14.29%	0.00%	0.00%	14
J	10.00%	0.00%	0.00%	10
K	66.67%	0.00%	0.00%	3
L	33.33%	0.00%	0.00%	3
U	22.50%	0.00%	0.00%	40

*: IN region of all HIV genotype data were obtained from LANL HIV database, and sequences encoding M50M, M50I, V151V, or V151I were extracted and calculated percentages of each population in each subtype. **: number of total sequences in each subtype.

## Supplementary Materials

The following are available online at https://www.mdpi.com/article/10.3390/v13112331/s1, Supplementary Methods: Supplementary methods for architecting a predicted structure of IN-RH fusion protein, Table S1: Samples profiles of HIV sequence, Table S2: Primer sequences, Table S3: Profile of sequence samples containing IN:M50I alone, Table S4: Summary of p24 concentration in HIV Stocks, Figure S1: Statistical analysis among VL and mutations, Figure S2: Predicted structure of IN-RH fusion protein.

## Abbreviations

ALLINIs	allosteric IN inhibitors
ART	anti-retroviral therapy
CA	capsid protein
DTG	dolutegravir
FBS	fetal bovine serum
GWAS	Genome-wide association study
HEPES	4-(2-hydroxyethyl)-1-piperazineethanesulfonic acid
HIV-1	human immunodeficiency virus type 1
IN	integrase
IN:M50I	methionine-to-isoleucine change at codon 50 of integrase
IN:S17N	serin-to-asparagine change at codon 17 of integrase
IN:V151	valine-to-isoleucine change at codon 151of integrase
LANL	Los Alamos National Laboratory
MA	matrix protein
mNL	modified cloned HIVNL4.3
NC	nucleocapsid protein
NGS	next generation sequence
NIAID	National Institute of Allergy and Infectious Diseases
NOPs	naturally occurring polymorphisms
PBMC	peripheral blood mononuclear cells
PHA	phytohemagglutinin
PR	protease
RH	RNaseH
RH:N79S	asparagine-to-serin change at codon 79 of RNaseH
RP10	RPMI-1640 supplemented with 10% FBS
RT	reverse transcriptase
VL	virus load
WB	Western Blotting
WT	wild-type
