# Supplementary material for: A Combination of M50I and V151I Polymorphic Mutations in HIV-1 Subtype B Integrase Results in Defects in Autoprocessing
Source: Viruses. 2021 Nov 22;13(11):2331. doi: 10.3390/v13112331 (PMC8625782; doi:10.3390/v13112331)
Supplement: Supplementary file 1 [file viruses-13-02331-s001.zip › Figure S1.pdf]

Figure S1

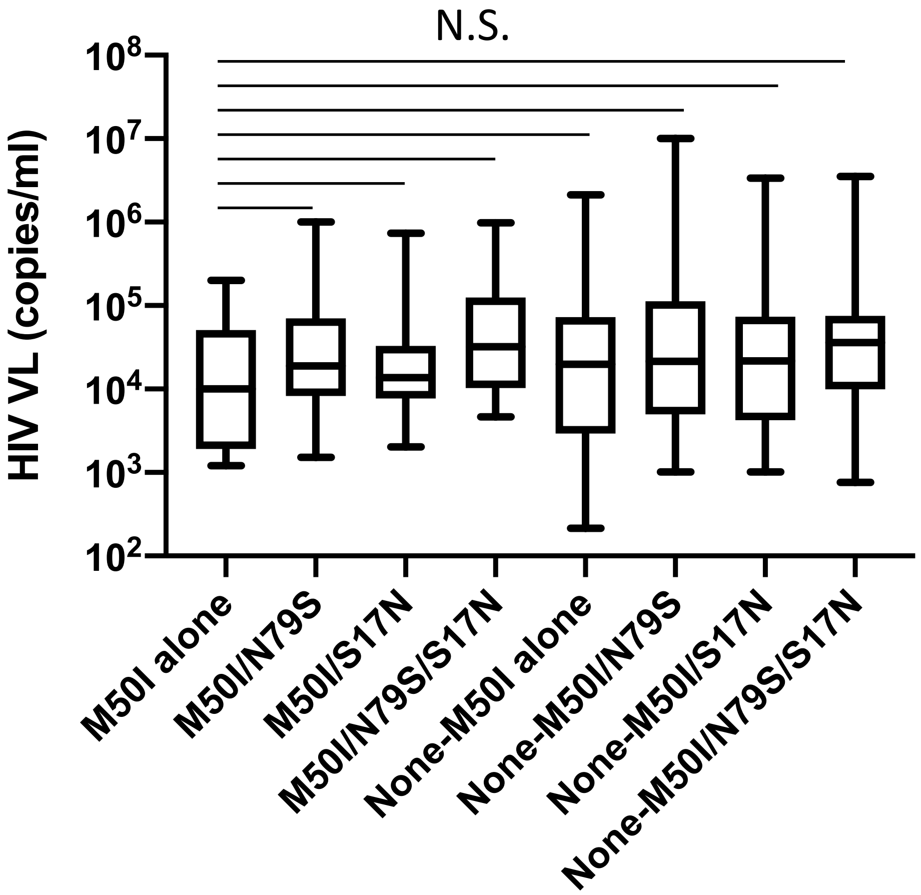

Figure S1. Statistical analysis among VL and mutations
To determine whether virus load (VL) in plasma and detected virus variants have a significant difference or not, we conducted the one-way ANOVA test between M50I alone and other combinations of mutations using a total of 529 samples.
None of the combinations showed significant differences in VL distribution with M50I mutation. N.S.: not significant ($p>0.05$)
