# Supplementary material for: A Combination of M50I and V151I Polymorphic Mutations in HIV-1 Subtype B Integrase Results in Defects in Autoprocessing
Source: Viruses. 2021 Nov 22;13(11):2331. doi: 10.3390/v13112331 (PMC8625782; doi:10.3390/v13112331)
Supplement: Supplementary file 1 [file viruses-13-02331-s001.zip › Figure S2.pdf]

**Figure S2**

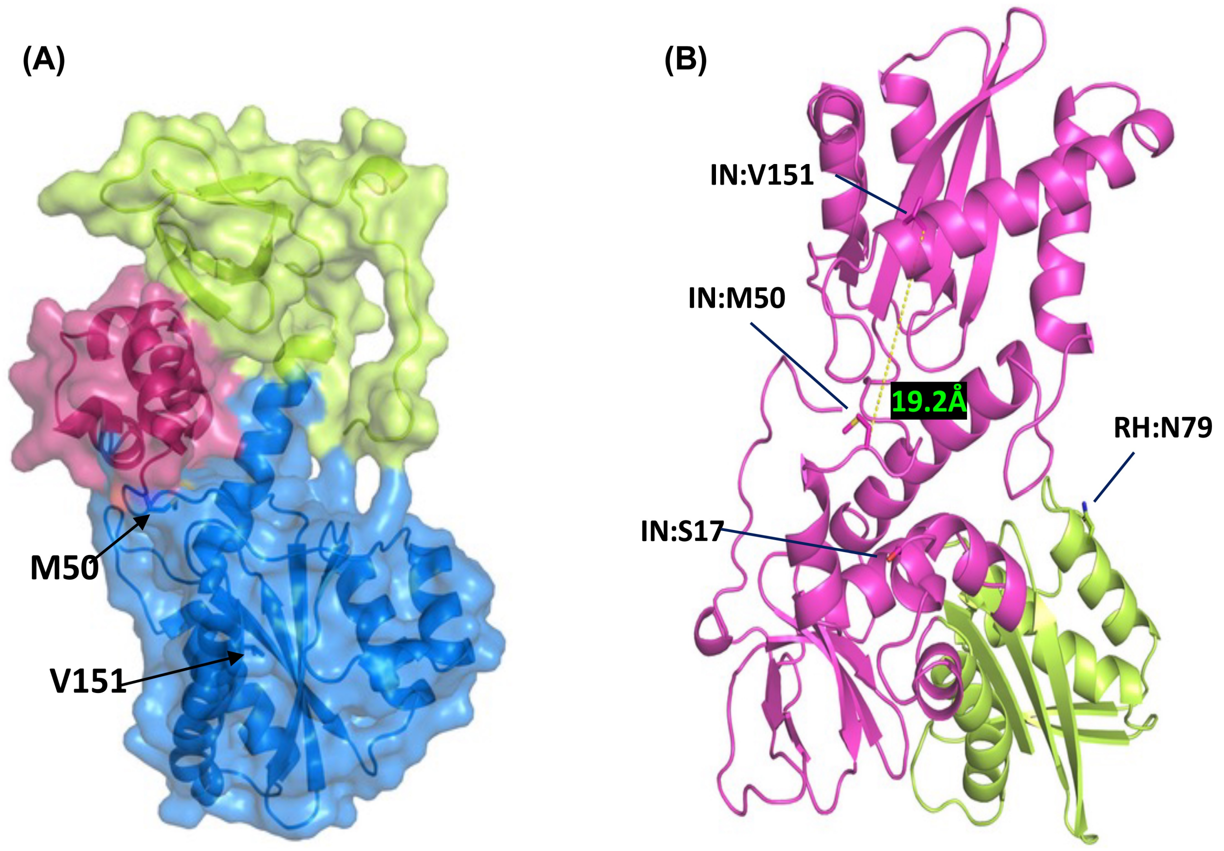


**Figure S2. Predicted structure of IN and IN-RT fusion proteins**

(A) Surface presentation of HIV-1 IN. The IN structure is illustrated in the surface model, with a transparency value of 0.5, where the N-terminal domain (NTD) (aa, 1-49) is colored in a warm pink, the catalytic core domain (CCD) (aa, 50-212) is colored in marine, and the C-terminal domain (CTD) (aa, 213-288) is color in limon. The side-chain residues of M50 and V151 are indicated in a stick. The figure was prepared in Open-Source software PyMOL. (B) Structure of *in silico* RH-IN (Wt) fusion protein. The *in silico* fusion protein was created as described in the Supplemental Method. IN and RH domains are indicated by magenta, and limon, respectively. Distance between IN:50 and IN:151 is indicated.
