# Supplementary material for: A Combination of M50I and V151I Polymorphic Mutations in HIV-1 Subtype B Integrase Results in Defects in Autoprocessing
Source: Viruses. 2021 Nov 22;13(11):2331. doi: 10.3390/v13112331 (PMC8625782; doi:10.3390/v13112331)
Supplement: Supplementary file 1 [file viruses-13-02331-s001.zip › sub.Modi.Table S3.pdf]

Table S3. A profile of sequence samples containing IN:M50I alone

Pt. ID [1]	Seq. ID [3]	Regimen [2]	Sample [4]	Blood Date [6]	VL [5]	Start Date [7]	End Date [8]	DOSE	
P1	1		Pre	4/28/17	201353				
		ABC/DTG/LAV				5/8/17		1 tab	Daily
P2		FTC/TFV-DF				12/1/08	1/1/15	100 mg/150 mg	Daily
		RAL				12/1/08	11/1/14	**400 mg**	**BID**
		DTG				11/1/14	1/1/15	**50 mg**	**Daily**
		ABC/DTG/LAV				1/1/15	5/11/17	**300 mg/600 mg/50 r**	**Daily**
	2		Post	6/8/17	50449				
	3		Pre	7/6/17	19832				
		ABC/DTG/LAV				7/10/17	3/3/19	300 mg/600 mg/50 n	Daily
		FTC/TFV-DF				7/10/17	7/10/17	100 mg/150 mg	Daily
		DTG				7/10/17	7/10/17	**50 mg**	**Daily**
	4		Post	3/28/19	12755				
	5		Pre	4/10/19	26508				
		ABC/DTG/LAV				4/17/19		300 mg/600 mg/50 n	Daily
P3	6	Never on ART	Pre	9/21/17	2044				
P4	7	BIC/TFV-AF/EM	On	12/11/18	51922	12/3/18		**1 tab**	**Daily**
P5	8	Never on ART	Pre	3/14/18	7261				
P6		ZDV				10/31/01	4/1/02	300 mg	bid
		LAV				10/31/01	4/1/02	150 mg	bid
		EFV				10/31/01	4/1/02	600 mg	At bedtime
		STV				6/7/02	6/1/04	40 mg	bid
		NFV				6/7/02	6/1/04	1250 mg	bid
		LPV/r				3/13/07	6/8/10	400mg/100mg	BID
		FTC/TFV-DF				3/13/07	3/29/17	200mg/ 300mg	daily
		AZV				6/8/10	3/29/17	300mg	Daily
		RTV				6/8/10	3/29/17	100mg	Daily
	9	EGV/COB/TAF/FTC	On	11/28/17	1207	3/30/17	6/14/18	1 tablet	Daily
	10		Pre	3/27/18	2886				
		BIC/TFV-AF/FTC				6/14/18		1 tablet	Daily
P7	11		Pre	7/24/07	52594				
		EFV/FTC/TVF-DF				10/28/09		1 tablet	Daily
P8*	12		Pre	11/28/17	3016				
	13		Pre	5/2/18	1502				
	14		Pre	11/14/18	1262				
		ABC/DTG/LAV				2/21/19	10/1/19	1 tablet	Daily
		ABC/DTG/LAV				1/10/20		1 tablet	Daily

1: Patients ID #
2: Sequence ID
3: Regimens, ABC: abacavir, AZV: Atazanavir, BIC: Bictegravir, COB: Cobicistat, DTG: dolutegravir, EFV: Efavirenz, EGV: Elvitegravir, FTC: Emtricitabine, LAV: lamivudine, LPV/r: Lopinavir/ritonavir, NFV: Nelfinavir, TFV-AF: tenofovir AF, TFV-DF: Tenofovir DF, RAL: Raltegravir, RTV: Ritonavir, STV: Stavudine, ZDV: Zidovudine
4: Status of the blood samples as Pre, Post, or ON therapy when collecting blood
5: Date of blood collection
6: Viral load, HIV RNA copy number (copies/ml)
7: Started date of new therapy
8: End of date of the therapy
*: The patient initially came to the NIAID clinic under the Long Term Non-Progressor protocol and moved to viremic patients under ART from 2019.
