# Supplementary material for: A Combination of M50I and V151I Polymorphic Mutations in HIV-1 Subtype B Integrase Results in Defects in Autoprocessing
Source: Viruses. 2021 Nov 22;13(11):2331. doi: 10.3390/v13112331 (PMC8625782; doi:10.3390/v13112331)
Supplement: Supplementary file 1 [file viruses-13-02331-s001.zip › Table S1.Samples profiles of HIV sequence.pdf]

Table S1. Samples profiles of HIV sequence

Sequence Time Points *	Pt No **	Sample No §
**1**	84	84
**2**	56	112
**3**	25	75
**4**	15	60
**5**	11	55
**6**	7	42
**7**	3	21
**8**	2	16
**9**	2	18
**10**	1	10
**11**	2	22
**14**	1	14
**Grand Total**	209	529

*: Frequency of sequence.

**: Patient numbers. All patients had>1000 copies /mL of viral load in plasma when sequence analysis was conducted, and none of HIV contained IN resistant mutations prior to IN inhibitor therapies.

§: Number of sequenced samples per frequency.
