# Supplementary material for: A Combination of M50I and V151I Polymorphic Mutations in HIV-1 Subtype B Integrase Results in Defects in Autoprocessing
Source: Viruses. 2021 Nov 22;13(11):2331. doi: 10.3390/v13112331 (PMC8625782; doi:10.3390/v13112331)
Supplement: Supplementary file 1 [file viruses-13-02331-s001.zip › Table S2_exS1.pdf]

Table S2: Primer sequences

| Primers 5'-3' | |
|---|---|
| E+ Primer Set * | |
| **Forward primer (HIV737U)** | 5′-GCGRCTGGTGAGTACGCC-3′ |
| **Reverse primer (GSM_5219_R)** | 5′-CCTAGTGGGATGTGTACTTCTGAAC-3′ |
| | |
| A4.5kb Primer Set | |
| **Forward primer (GSM_682_F)** | 5′-TCTCTCGACGCAGGACTCG-3′ |
| **Reverse primer (rt-in-2-R)** | 5′-CCTAGTGGGATGTGTACTTCTGAACTTA-3′ |
| | |
| Point Mutagenesis Primers | |
| **I151V S** | 5′-CAATCCCCAAAGTCAAGGAGTAGTAGAATCTATGAATAAAG-3′ |
| **I151V AS** | 5′-CTTTATTCATAGATTCTACTACTCCTTGACTTTGGGGATTG-3′ |

*: primer sequences for RT-PCR
