# Supplementary material for: A Combination of M50I and V151I Polymorphic Mutations in HIV-1 Subtype B Integrase Results in Defects in Autoprocessing
Source: Viruses. 2021 Nov 22;13(11):2331. doi: 10.3390/v13112331 (PMC8625782; doi:10.3390/v13112331)
Supplement: Supplementary file 1 [file viruses-13-02331-s001.zip › Table S4.Profiles of p24 concentration in HIV Stocks.pdf]

Table S4. Summary of p24 concentration in HIV Stocks

Phenotype *	p24 (μg/mL) **	% †	p value ‡
WT	110.0 ± 22.7	100	N/A
M50I	93.5 ± 30.2	85	p>0.05
V151I	118.3 ± 38.9	107	p>0.05
M50I/V151I	0.3 ± 0.1	0.3	P<0.01

*: Phenotype of HIV Stock; WT: wild type HIV, M50I: HIV carrying IN:M50I, V151I: HIV carrying IN:V151I, M50I/V151I: HIV carrying IN:M50I/V151I.

**: p24 concentrations in HIV stocks. Data indicates Means ± SE, (n=4).

†: Percentage of stock concentration of each stock compared to that of WT

‡: p value from unpaired Student *t* test of each stock compared to WT.
