# Supplementary figures and images for: A Combination of M50I and V151I Polymorphic Mutations in HIV-1 Subtype B Integrase Results in Defects in Autoprocessing
Source: Viruses. 2021 Nov 22;13(11):2331. doi: 10.3390/v13112331 (PMC8625782; doi:10.3390/v13112331)

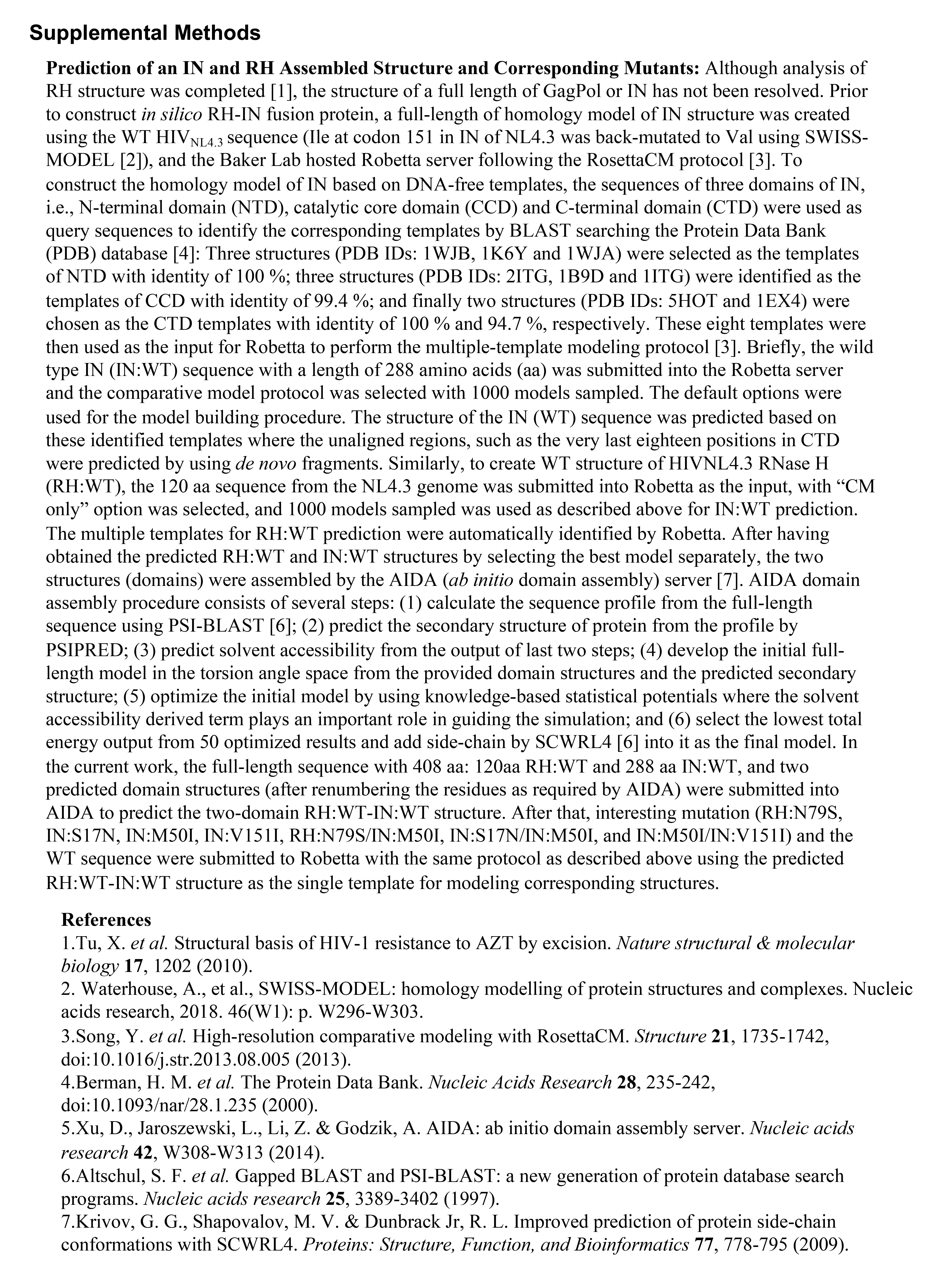

Supplement: Supplementary file 1 [file viruses-13-02331-s001.zip › S. materials_S.Method_tif.jpg]
